# Meetings of Societies

**Published:** 1931

**Authors:** 


### Meetings of Societies

### Bristol Medico-Chirurgical Society

The second meeting of the session was held at the University on 12th November, 1930, at 8.30 p.m. The President was in the Chair.

Mr. R. S. STATHAM gave an address on "**Ectopic Gestation.**"

The President pointed out that the subject was one of great importance to the general practitioner. He is the one who first sees the case, and as the time factor is so important much depends upon an early correct diagnosis. He was of the opinion that in the operation speed is of prime importance, and he agreed that as much as possible of the extravasated blood should be removed. It causes troublesome post-operative distension if left.

Dr. ROWLEY quoted a case where the first symptom was pain in the coccyx, and he wondered if anyone else had made the same observation.

Mr. RENDLE SHORT said the subject was of interest to the general surgeon as he often operated upon these cases. He agreed that as soon as the diagnosis was made the patient should be subjected to operation. He quoted one case in which the patient bled to death in four minutes after the rupture of the tube.

Drs. STOCK and FLEMMING had both noted that in these cases as soon as the bleeding was stopped the pulse very rapidly fell to normal.

Dr. H. ROGERS asked the speaker if he had had any experience with the Ascheim-Zondek test for the differential diagnosis in these cases.

Drs. ALEXANDER and LILY BAKER and Mr. GRIFFITHS quoted cases.

Mr. STATHAM in replying said that he had had no experience with the Ascheim-Zondek test, and thought that it would only lead to loss of valuable time. He remembered Dr. ROWLEY'S case, but it was the only one in which he had noted this peculiar symptom. He thought that the measure of the pulse-rate was due to the speed at which the blood was lost, but as soon as this was stopped the rate rapidly fell again.

---

The third meeting of the session was held at the University on 10th December, 1930. The President was in the Chair.

Dr. ALEXANDER showed and described a case of **Striæ Atrophicæ,** and Mr. G. B. BUSH a number of X-rays demonstrating **Silicosis of the Lung.**

Dr. CAREY F. COOMBS showed and described a series of sections from the auriculo-ventricular bundle, normal and abnormal; also diagrams illustrating the pathology of the chief cardiac infections.

Dr. HERBERT ROGERS showed and described specimens illustrating **Necrosis of the Myocardium.** A paper based on these appears in the current issue.

Dr. BRUCE PERRY gave a paper on "**Congenital Heart Disease as seen in Elementary School Children,**" illustrated by lantern slides. This paper appears in the current issue.

Dr. C. E. K. HERAPATH stressed the importance of recognizing the signs of a patent inter-ventricular septum and of realizing that this condition entailed no cardiac disability.

Dr. RICHARD CLARKE said that he had found that the localization of the physical signs in congenital heart disease varied from one examination to the next. He thought that the alleged "heart attacks" were of no significance. He described how latent cyanosis could be made more obvious by placing a limb in a hot bath. He stated that of the cases of congenital heart disease which he had seen in infancy only one-third had survived.

Dr. TAYLOR asked the fate of the cases of congenital heart disease, and suggested that if the figures given were correct the condition should be found more often post-mortem. Referring to Dr. Rogers's paper, he thought it was very difficult to be sure that the condition he described as an infarct of the auricle was not actually an infective condition.

Dr. A. F. ALFORD described a "heart attack" which he had observed personally in a child with a congenital malformation of the heart, and stated that it was indistinguishable from an attack of "petit mal."

Mr. JACKMAN thanked Dr. Coombs for showing his simple and easily comprehended diagrams of cardiac pathology.

Drs. COOMBS, ROGERS and PERRY replied.

---

The fourth meeting of the session was held at the University on 14th January, 1931. The President was in the Chair.

Dr. MAYES showed a number of X-rays.

Dr. A. L. TAYLOR showed and described lantern slides, illustrating silicosis and asbestosis of the lung.

Dr. NORMAN BURGESS showed and described two microscopic specimens.

Mr. A. W. ADAMS gave a paper on "**Spinal Anæsthesia.**"

The PRESIDENT, Dr. GORNALL, Mr. KEMM, Mr. WOOD, Mr. GRIFFITHS, Mr. STOCK and Dr. CORFIELD discussed the pap er.

---

The fifth meeting of the session was held at the University on 11th February, 1931. The President was in the Chair.

Sir EWEN J. MACLEAN gave an address entitled "**Some Reminiscences and a Forecast.**" This will appear in our next issue.

The speaker indicated that he wished his paper to be followed by a discussion; those who took part were Mr. WRIGHT, Mr. WALTERS, Dr. ALEXANDER, Col. LISTER and Dr. PARRY.

Sir EWEN MACLEAN replied.

The sixth meeting of the session was held at the University on 11th March, 1931. The President was in the Chair.

Dr. A. L. TAYLOR showed and described pathological specimens and Mr. WANSBOROUGH a number of X-ray films.

Dr. H. H. CARLETON then gave a paper on "**Some Disorders of Movement and their Mechanism,**" illustrated by lantern slides. This will appear in our next issue.

Dr. BLACHFORD, Dr. GORDON, Dr. F. BODMAN and Dr. KENNEDY discussed the paper, and Dr. CARLETON replied.

